# Opiate Analgesics as Negative Modulators of Adult Hippocampal Neurogenesis: Potential Implications in Clinical Practice

**DOI:** 10.3389/fphar.2017.00254

**Published:** 2017-05-09

**Authors:** Valeria Bortolotto, Mariagrazia Grilli

**Affiliations:** Laboratory of Neuroplasticity, Department of Pharmaceutical Sciences, University of Piemonte OrientaleNovara, Italy

**Keywords:** opiates, morphine, methadone, tapentadol, neural stem cells, adult neurogenesis, chronic pain, depression

## Abstract

During the past decade, studies of the mechanisms and functional implications of adult hippocampal neurogenesis (ahNG) have significantly progressed. At present, it is proposed that adult born neurons may contribute to a variety of hippocampal-related functions, including specific cognitive aspects and mood regulation. Several groups focussed on the factors that regulate proliferation and fate determination of adult neural stem/progenitor cells (NSC/NPC), including clinically relevant drugs. Opiates were the first drugs shown to negatively impact neurogenesis in the adult mammalian hippocampus. Since that initial report, a vast array of information has been collected on the effect of opiate drugs, by either modulating proliferation of stem/progenitor cells or interfering with differentiation, maturation and survival of adult born neurons. The goal of this review is to critically revise the present state of knowledge on the effect of opiate drugs on the different developmental stages of ahNG, as well as the possible underlying mechanisms. We will also highlight the potential impact of deregulated hippocampal neurogenesis on patients undergoing chronic opiate treatment. Finally, we will discuss the differences in the negative impact on ahNG among clinically relevant opiate drugs, an aspect that may be potentially taken into account to avoid long-term deregulation of neural plasticity and its associated functions in the clinical practice.

## Adult Neurogenesis: Relevance in Physiology, Pathology, and Therapy

Extensive experimental work demonstrated that new neurons can be generated in adult mammalian brain (Bergmann et al., 2015). One adult neurogenic area is the dentate gyrus, in the hippocampal formation. Here, in a subregion called the SubGranular Zone (SGZ), resident neural stem/progenitor cells (NSC/NPC) are present, can self-renew and give rise to transiently amplifying progenitor cells which, in turn, generate neuroblasts capable of terminal neuronal differentiation in the granular cell layer (Kempermann et al., 2003, 2004; Seri et al., 2004; Bonaguidi et al., 2012).

Recently a comprehensive review summarized data on how hippocampal function and related behavior may be modified by newborn neurons (Gonçalves et al., 2016). At present, adding new neurons in the hippocampal circuitry is suggested to result in encoding of temporal information into memories and in cognitive flexibility during new task learning. Pattern separation is the process that reduces overlap in the representation of similar memories and adult hippocampal neurogenesis (ahNG) has been associated with improved performance in pattern separation behavioral tasks (Aimone et al., 2009; Sahay et al., 2011). In several studies a reduction in newborn cells was found to correlate with specific cognitive deficits, and in particular with spatial memory impairment (Shors et al., 2001; Aimone et al., 2006; Denis-Donini et al., 2008; Deng et al., 2010; Couillard-Despres et al., 2011; Aimone et al., 2014). Context-dependent memory, and specifically performance in contextual fear conditioning tasks, was also found to be neurogenesis-dependent (Gonçalves et al., 2016).

A vast array of research investigated factors that regulate ahNG. External stimuli, including learning, environmental enrichment, and exercise have profound effects on proliferation/ differentiation of NPC as well as survival of their progeny (van Praag et al., 2005; Garthe et al., 2016). Aging, chronic stress and social isolation result in negative impact on ahNG (Schoenfeld and Gould, 2012; Seib and Martin-Villalba, 2015; Famitafreshi et al., 2016) and reduction of hippocampal neurogenesis has been hypothesized to contribute to cognitive decline or mood alterations associated with those conditions (Aimone et al., 2014).

Extensive research efforts suggest that ahNG may be deregulated in several neuropsychiatric disorders, including major depressive disorder (Kempermann and Kronenberg, 2003; Mirescu and Gould, 2006; Denis-Donini et al., 2008; Kempermann et al., 2008; Yun et al., 2016).

An intringuing aspect of ahNG is its susceptibility to pharmacological modulation (Duman et al., 2001; Dranovsky and Hen, 2006; Xu et al., 2006; Zhao et al., 2008; Bortolotto et al., 2014). Antidepressants increase hippocampal neurogenesis (Malberg, 2004). Interestingly, such increase requires several weeks and occurs in parallel with the onset of the antidepressant effects (Mendez-David et al., 2013). A vast array of experimental studies demonstrated that antidepressants counteract stress-reduced ahNG in rodent models of depressive-like disorder (Pittenger and Duman, 2008; Eisch and Petrik, 2012). An increased number of hippocampal NPC was reported in postmortem brain of depressed patients who were treated with antidepressants (Boldrini et al., 2009, 2012, 2014). Although still controversial, it has been suggested that antidepressants require ahNG to exert some behavioral effects in rodents (Santarelli et al., 2003; David et al., 2009) and that ahNG may contribute to the antidepressant activity of these drugs in the clinical setting (Jun et al., 2012). In line with this hypothesis, preclinical studies evaluating effects on ahNG have become part of the discovery process of recently approved antidepressant drugs (Soumier et al., 2009; Guilloux et al., 2013) or of clinically relevant drugs which, in addition to their approved indications, may potentially exert antidepressant activity (Valente et al., 2012; Cuccurazzu et al., 2013).

## Opiates as Negative Modulators of ahNG

Emerging evidence suggest that several psychoactive drugs result in molecular changes that may negatively affect different aspects of ahNG (Eisch et al., 2000; Yamaguchi et al., 2004). These findings have important clinical implications since they raise the possibility that cognitive dysfunction and/or mood alteration in the setting of such drug use and/or abuse may, at least in part, be related to alterations in ahNG (Yun et al., 2016).

Opiate drugs are powerful analgesics which are also among the most commonly abused addictive drugs. Clinical research suggested the occurrence of deficits in memory tasks, attention, verbal fluency and general cognitive performance in opiate addicts (Cipolli and Galliani, 1987; Guerra et al., 1987; Gruber et al., 2007). Controversial, but of potential clinical relevance, is the possibility that cognitive impairment may also occur in non-addicted patients subjected to chronic opiate treatment (Sjøgren et al., 2005; Gaertner et al., 2006; Kendall et al., 2010; Kurita et al., 2011; Højsted et al., 2012). Despite opiate effects on cognition are difficult to decipher due to their central depressant effects, preclinical research supports the idea that chronic administration of opiates may interfere with cognition independently of performance (Spain and Newsom, 1991; Miladi Gorji et al., 2008). As an example, in rats, chronic morphine administration impaired hippocampal dependent long-term memory retention. Moreover, a selective impairment in spatial memory, which is considered to be neurogenesis-dependent, was observed in morphine- compared to vehicle-treated animals (Miladi Gorji et al., 2008).

Depression often co-occurs with chronic pain and an emerging line of inquiry is also the association between opiate use and the risk of depression (Scherrer et al., 2014, 2015, 2016; Smith et al., 2015). In particular, the onset of a new depressive state has been associated with longer drug administration (Scherrer et al., 2016).

Altogether several experimental data suggest that long-term opiate may produce maladaptive changes in brain structures involved in cognition and mood regulation such as the hippocampus. In morphine-dependent animals, mechanisms responsible for the disruption of long-term memory retention have been suggested, including effects on dendritic spine stability (Liao et al., 2005) and long-term potentiation (Nugent et al., 2007). ahNG is among the forms of neural plasticity which are regulated by opiates (Zhang et al., 2016a). Actually, opiates were the first drugs shown to negatively impact hippocampal neurogenesis (Eisch et al., 2000). Since then, numerous rodent studies confirmed that finding (Mandyam et al., 2004; Kahn et al., 2005; Eisch and Harburg, 2006; Arguello et al., 2008; Zhang et al., 2016b). Based on these reports, it can be hypothesized that opiates may produce long-lasting effects on the neuronal circuitry involved in mood and cognition through, at least in part, disruption of ahNG.

## Opiate Effect on Distinct Stages and Cell Types of ahNG

Adult neurogenesis is a complex, multistage process which consists of a series of developmental events, namely proliferation, differentiation, migration, maturation and survival of NSC/NPC and their progeny. Based on their developmental stage, different cells participating in ahNG can be recognized and classified. Briefly, radial-glia-like stem cells, which are both glial fibrillary acidic protein (GFAP) and nestin positive are defined as Type-1 cells; Type-2 cells are neural progenitors which are highly proliferative and nestin^+^/GFAP^-^; neuroblasts which express doublecortin and the polysialylated form of neural cell adhesion molecule (PSA-NCAM) are referred to as Type-3 cells.

Over the last few years, extensive progress has been made on how morphine can disrupt neurogenesis in the adult rodent hippocampus (Zhang et al., 2016a). The first report correlating opiate administration and adult neurogenesis was by the group of Amelia Eisch that, in 2000, reported that chronic morphine, administered via a subcutaneous pellet, decreased the number of cells which incorporated the thymidine analog BromodeoxyUridine (BrdU) in rodent SGZ. Importantly, no effect of acute morphine administration was reported (Eisch et al., 2000). Since then, extensive evidence has been accumulated on the negative impact of morphine on NSC/NPC proliferation, using BrdU or endogenous markers of cell proliferation/cell cycle (Mandyam et al., 2004; Kahn et al., 2005; Arguello et al., 2008). Morphine effects appeared to be independent from the route of administration (subcutaneous pellet vs. intraperitoneal injection) and to occur during settings of both forced and self-administration (Mandyam et al., 2004; Kahn et al., 2005; Fischer et al., 2008).

Survival of newborn cells is also reduced by morphine. After being born in the dentate gyrus, a large portion of newly generated cells die within a few days (Dayer et al., 2003). Chronic morphine treatment decreased granule cell survival *in vivo*, by largely reducing the number of 4-week-old BrdU-labeled cells in the dentate gyrus of drug-treated compared to control rats (Eisch et al., 2000). Although adult hippocampal NPC can be isolated and maintained in culture with self renewal and multipotential properties (Valente et al., 2012; Cuccurazzu et al., 2013), surprisingly few studies investigated the effects of opiates *in vitro*. In one report morphine exposure in cultured NSC/NPCs resulted not only in reduced proliferation but also in an increased caspase-3 activity in nestin^+^/GFAP^+^ cells and not in neuroblasts (Willner et al., 2014).

The detailed mechanisms of regulation of ahNG by morphine remain to be fully clarified. There is evidence of morphine acting directly on neural progenitors by engagement of opioid receptors on their cell surface, but also indirectly. Opiate analgesics exert their effects through receptor subtypes, referred to MOR, KOR and DOR, which interact with endogenous opioid peptides. The contribution of different receptor subtypes to the negative effects on ahNG has been investigated by both genetic and pharmacological approaches, pointing to a major role of MOR. We demonstrated that morphine adversely impacted on neuronal differentiation, neurite outgrowth and survival of adult hippocampal NPC and their progeny (Meneghini et al., 2014). MOR pharmacological blockade confirmed that the receptor subtype was responsible for morphine effects (Meneghini et al., 2014). Interestingly, knock-out of MOR enhanced adult-born granule cell survival *in vivo*, suggesting that endogenous opioids may have a negative effect on ahNG (Harburg et al., 2007). These findings are not consistently confirmed *in vitro*. In isolated rat hippocampal NPC, incubation with β-endorphin increased total DNA content and the number of cells expressing proliferation markers such as Proliferating Cell Nuclear Antigen (PCNA) and phosphorylated histone H3 (pHisH3), and these effects were antagonized by naloxone (Persson et al., 2003a). The same research group reported that MOR and DOR antagonists decrease proliferation of cultured NPC (Persson et al., 2003b). Similarly, the long acting opioid antagonist naltrexone was shown to reduce cell proliferation in the hippocampus of adult rats (Holmes and Galea, 2002).

Opiates also interfere with NPC fate specification. Type-3 cell number was diminished by chronic morphine (Kahn et al., 2005). Moreover, in rat, repeated morphine treatment altered the GABAergic phenotype of adult-born hippocampal granule cells by increasing the GABA synthesizing enzyme glutamate decarboxylase-67 (Kahn et al., 2005). A more detailed analysis using BrdU and Ki67 proliferation markers concluded that morphine treatment increased Type-2b and decreased Type-3 cells in mouse SGZ (Arguello et al., 2008). Also *in vitro* a remarkable decrease in neuronal differentiation of mouse hippocampal NPC by morphine has been demonstrated, an effect which is MOR mediated (Meneghini et al., 2014; Willner et al., 2014). Chronic treatment with MOR and DOR antagonists decreased adult NSC/NPC differentiation into astrocytes and oligodendrocytes, while favoring their neuronal differentiation. In the same experimental setting KOR antagonists had no effect (Persson et al., 2003b). It cannot be excluded that opiates may also affect astrocytes (that express opioid receptors) which, in turn, can modulate ahNG with different mechanisms, including via secreted molecules (Cvijetic et al., 2017). Immature adult generated neurons are excited by GABA (Ge et al., 2006) and they need excitatory signals from the preexisting circuit to complete their differentiation and maturation. Opiate agonists may interfere with this process by decreasing GABA release (Neumaier et al., 1988). Studies are needed to further understand the role of endogenous opioids and receptors in ahNG homeostasis (Lutz and Kieffer, 2013).

The intracellular signaling pathways involved in the negative effects of morphine on neural progenitors were investigated only in a few studies. The basic helix-loop-helix transcription factor NeuroD1 is negatively regulated by morphine in NPC cultures (Zheng et al., 2010). Under the conditioned place preference paradigm morphine, through a mechanism involving NeuroD1, impaired the differentiation of NSC/NPC into immature neurons (Zhang et al., 2016b). Xu et al. (2015) demonstrated that, in presence of morphine, mouse NPC preferentially differentiated into astrocytes and not neurons. This effect was mediated by MOR and by miR-181a/Prox1/Notch1 pathway activation. Interestingly, the same group also demonstrated that miR-181a/Prox1/Notch1 pathway regulates NPC differentiation in a ligand-dependent manner (Xu et al., 2015), pointing to differences in the effect of distinct opiate molecules on mouse NPC differentiation. Morphine modulates the lineage-specific differentiation of NPC by PKC𝜀-dependent ERK activation with subsequent TAR RNA-binding protein (TRBP, a cofactor of Dicer) phosphorylation and miR-181a maturation. Conversely fentanyl activated ERK via the β-arrestin-dependent pathway, followed by nuclear translocation of phosphoERK.

Overall, available data support the idea that morphine negatively affects neurogenesis acting on multiple cellular types and stages of the neuroplasticity process. Morphine properties on neurogenesis are also shared by other opiates. The partial agonist buprenorphine, when administered via subcutaneous injections over a 3-day period, reduced the number of actively proliferating cells in the hippocampus of adult mice (Pettit et al., 2012). On the other hand, differences in the signaling pathways activated in NPC by different opiate drugs may underlie potential differences in their impact on ahNG.

In the future studies, should be specifically designed to correlate more stringently the disruptive cognitive effects of distinct opiates with specific alterations in ahNG and to discriminate those that are strictly dependent on neurogenesis from the ones that are neurogenesis-independent.

## Not All Opiates are Created Equal: Different Impact on Hippocampal Neurogenesis of Distinct Drugs

A recent *in vivo* study in rat found that chronically administered methadone does not alter parameters relevant to ahNG including the number of Ki67-, doublecortin-, or BrdU-immunoreactive cells (Sankararaman et al., 2012). These results suggest that, unlike morphine, methadone may not alter hippocampal plasticity. Interestingly methadone is an atypical opiate, since it is a MOR agonist and a non-competitive NMDA antagonist (Gorman et al., 1997). Incidentally, NMDA antagonists positively modulate hippocampal neurogenesis in rodents (Nacher et al., 2001; Maekawa et al., 2009). Future investigation should address whether the lack of negative effects on ahNG by methadone could be ascribed to its NMDA receptor antagonism.

Tapentadol is a centrally acting analgesic drug which combines, in a single molecule, MOR agonism and blockade of norepinephrine (NE) reuptake (Tzschentke et al., 2007). Since ahNG is positively modulated by NE (Jha et al., 2006; Kulkarni and Dhir, 2007), we compared the effects of morphine and tapentadol on differentiation, maturation and survival of neurons generated from adult hippocampal NPC *in vitro*. Morphine significantly hampered neuronal differentiation, neurite outgrowth and survival of adult NPC and their progeny (Meneghini et al., 2014). In presence of tapentadol cell survival was not affected. Conversely, tapentadol reduced the number of newly generated neurons and their neurite outgrowth but only at concentrations which activate MOR and not at higher ones which block NE reuptake. Specifically, tapentadol proneurogenic and antiapoptotic effects appeared to be mediated by activation of β2 and α2 adrenergic receptors, respectively (Meneghini et al., 2014). As a proof of concept, in the same experimental model, morphine antineurogenic and proapoptotic effects were counteracted by reboxetine, an antidepressant which selectively blocks noradrenaline reuptake (Meneghini et al., 2014). In line with these *in vitro* results, chronic administration of a clinically relevant dose of tapentadol did not negatively affect proliferation and differentiation toward the neuronal lineage of newly generated cells in adult mouse hippocampus (Meneghini et al., 2014). Altogether these data support the idea that the noradrenergic component in tapentadol has the potential to counteract the adverse MOR-mediated effects on hippocampal neurogenesis both *in vitro* and *in vivo*. In principle, this counter-balancing effect may result, like for the atypical opiate methadone, in less or no dysfunction in adult neurogenesis and, potentially, in neurogenesis-associated functions after long-term treatment *in vivo*.

## Clinical Implications and Long-Term Perspectives

Cognitive dysfunction has been often reported in opiate drug abusers (Eisch and Harburg, 2006; Canales, 2007). Moreover, major depression represents an important comorbidity in chronic pain states and recent work has suggested an association between prolonged opiate use and the risk of new onset depression (Scherrer et al., 2015, 2016).

Preclinical evidence summarized in this review support the idea that disruption of ahNG may, at least in part, contribute to the long-lasting effects produced by some opiates, like morphine, on cognition and mood regulation. Although preliminary, these preclinical data mark the importance of taking into account inhibition of ahNG as a potential long-term consequence of opiate use also in the clinical setting. This aspect appears even more relevant in consideration of the fact that chronic pain *per se* may induce profound changes in hippocampal plasticity, including deregulated hippocampal neurogenesis (Mutso et al., 2012; Dellarole et al., 2014).

While we wait for a definitive answer on any causal correlation between long term-opiate treatment and cognitive and emotional impairment in chronic pain patients, preclinical studies should be undertaken to increase our current understanding of the cellular and molecular effects of opiates on adult NPC and their progeny. Moreover, experimental work should be aimed at understanding differences between distinct opiates in their potential for disrupting ahNG. Ultimately these studies may help us to unravel different level of risk associated with distinct treatment options. In principle, drugs like methadone and tapentadol may result, after drug long-term treatment, in reduced or absent dysfunction in ahNG compared to morphine and other opiates. Alternatively, other analgesic drugs which do not reduce but rather increase ahNG in preclinical models like pregabalin/gabapentin (Valente et al., 2012) and acetyl-L-carnitine (Cuccurazzu et al., 2013) could be proposed, at least in neuropathic pain where they are effective. A better understanding of whether different opiate molecules affect molecular and cellular aspects of ahNG may also help designing and developing novel analgesic drugs for chronic pain states. Several drugs acting at different targets are under development for chronic pain treatment (Sasikumar et al., 2010; Bennett et al., 2012; Chang et al., 2015; Yaksh et al., 2015). We propose that future drug design should take into consideration the development of powerful analgesics that, by preserving ahNG, may also protect cognitive functions and counteract mood alterations which often represent comorbidities in chronic pain states.

## Author Contributions

VB and MG were involved in the concept, literature screening, and writing of the article, and approved it for publication.

## Conflict of Interest Statement

In the past, MG has received research grants from drug companies manufacturing analgesic drugs, including Angelini S.p.A, Grunenthal GmbH, Pfizer, Sigma Tau. The other author declares that the research was conducted in the absence of any commercial or financial relationships that could be construed as a potential conflict of interest.
